# Evaluating a Nationwide Recreational Football Intervention: Recruitment, Attendance, Adherence, Exercise Intensity, and Health Effects

**DOI:** 10.1155/2016/7231545

**Published:** 2016-06-29

**Authors:** Liljan av Fløtum, Laila S. Ottesen, Peter Krustrup, Magni Mohr

**Affiliations:** ^1^Faculty of Natural and Health Sciences, University of the Faroe Islands, 100 Tórshavn, Faroe Islands; ^2^Department of Nutrition, Exercise and Sports, Copenhagen Centre for Team Sport and Health, University of Copenhagen, 2100 Copenhagen, Denmark; ^3^Sport and Health Sciences, College of Life and Environmental Sciences, University of Exeter, St. Luke's Campus, EX1 2LU Exeter, UK; ^4^Center for Health and Human Performance, Food and Nutrition, and Sport Science, University of Gothenburg, 40530 Gothenburg, Sweden

## Abstract

The present study evaluated a nationwide exercise intervention with Football Fitness in a small-scale society. In all, 741 adult participants (20–72 yrs) were successfully recruited for Football Fitness training in local football clubs, corresponding to 2.1% of the adult population. A preintervention test battery including resting heart rate (RHR), blood pressure, and body mass measurements along with performance tests (Yo-Yo Intermittent Endurance level 1 (Yo-Yo IE1), the Arrowhead Agility Test, and the Flamingo Balance Test) were performed (*n* = 502). Training attendance (*n* = 310) was 1.6 ± 0.2 sessions per week (range: 0.6–2.9), corresponding to 28.8 ± 1.0 sessions during the 18 wk intervention period. After 18 wks mean arterial pressure (MAP) was −2.7 ± 0.7 mmHg lower (*P* < 0.05; *n* = 151) with even greater (*P* < 0.05) reductions for those with baseline MAP values >99 mmHg (−5.6 ± 1.5 mmHg; *n* = 50). RHR was lowered (*P* < 0.05) by 6 bpm after intervention (77 ± 1 to 71 ± 1 bpm). Yo-Yo IE1 performance increased by 41% (540 ± 27 to 752 ± 45 m), while agility and postural balance were improved (*P* < 0.05) by ~6 and ~45%, respectively. In conclusion, Football Fitness was shown to be a successful health-promoting nationwide training intervention for adult participants with an extraordinary recruitment, a high attendance rate, moderate adherence, high exercise intensity, and marked benefits in cardiovascular health profile and fitness.

## 1. Introduction

In the past decade, recreational football has been shown to be an effective training method that provides a broad-spectrum health response [1]. The vast majority of the studies testing the effects of football training on health and fitness are randomized controlled trials on subject groups of both genders [1, 2], different age groups [2, 3], and groups of patients suffering from hypertension [4, 5], type 2 diabetes [6, 7], and cancer [8, 9]. Moreover, the health impact of football training has been compared to running [1, 10], strength training [2, 11], swimming [5, 12], dancing [13], and vibration training [14] with overall greater effects of football training. A recent meta-analysis and systematic review of the fitness and health effects of sports disciplines concluded that most evidence was found for football and running, especially with regard to cardiovascular and metabolic fitness [15]. Accordingly, another recent meta-analysis and systematic review of the effects of recreational football on aerobic fitness concluded that short-term intervention produced marked positive responses to maximal oxygen uptake, with average increases of 3.51 mL/min/kg [16].

Scientific evidence demonstrates that physical inactivity increases the risk of several adverse health conditions, including major noncommunicable diseases such as coronary heart disease, type 2 diabetes, and different types of cancers, and shortens life expectancy [17]. According to a recent report by Lee et al. [17], it is estimated worldwide that physical inactivity causes 6% of the burden of disease from coronary heart disease, 7% of type 2 diabetes, and 10% of breast and colon cancer. Moreover, inactivity causes 9% of premature mortality, corresponding to 5.3 million of the 57 million deaths that occurred worldwide in 2008. If inactivity was lowered by 10% or 25%, more than 533,000 or more than 1.3 million deaths, respectively, could be averted yearly [17]. Women experience a decline in health status in the years of menopause [18], including a higher prevalence of cardiovascular diseases, type 2 diabetes, and impaired muscle and bone health [19, 20]. Thus, middle-aged women appear to be a critical population group in relation to lifestyle diseases. Since exercise exerts a preventive effect on development of lifestyle-related deficiencies [21], exercise interventions targeting a broad health response are highly relevant for middle-aged women close to the menopause. Since much of the world's population is inactive, the association with noncommunicable diseases presents a major public health issue. Thus, it is a worldwide challenge to provide initiatives that will get inactive people to become active and increase life expectancy.

In Denmark the Football Fitness concept is being used as an instrument of health policy with the aim of bringing more adult members into the football clubs and producing more physically active adults. Considering the beneficial position of the organized sport especially in Scandinavia [22] highlights the importance of determining the most effective programs that sporting organizations can use to increase adult participation in physical activity. Ooms et al. [23] state that further research is required to examine factors influencing the long term sustainability of health enhancing physical activity programs in the organized sports setting. The design of the Danish Football Fitness concept differs from the traditional Scandinavian recreational club football due to the following characteristics: there is no tournament structure, there is a reduced membership fee, and the training is a mean in itself and not obligatory. The communication on the team is mostly going on at Facebook or some other social media sites. Although FF is marketed as a health or fitness initiative, the Danish FA is constantly emphasizing that it is also about pleasure and friendship, for example, by adding the subhead “Fun, Fitness, and Friendship” and this is also what the participants in Denmark stress is important for them together with the feeling of flexibility that the design allows for [24]. However, it is not known how efficient the Football Fitness concept is when initiated as a nationwide intervention involving football training in local football clubs. In the Faroe Islands, a Northern European country with only ~50,000 inhabitants, football is a popular sport and the country has the world's highest number of active football players per capita. Nationwide exercise intervention studies are well suited to small-scale societies such as the Faroe Islands due to the short communication infrastructure and geographical distances.

The purpose of the study was to evaluate the recruitment, attendance rate, adherence, exercise intensity, and intervention-induced effects on cardiovascular health profile and fitness of a nationwide exercise intervention involving Football Fitness in local football clubs. We hypothesized that football training for adult Faroese participants would elicit high attendance rates, high exercise intensities, and large cardiovascular fitness and health effects over 18-week intervention period.

## 2. Materials and Methods

### 2.1. Subjects

741 participants were originally recruited to a national organized football intervention in the Faroe Islands, corresponding to ~1.5% of the total population (~50,000) or 2.1% of the total population older than 19 yrs. Of the total sample, 502 subjects (age 42.2 ± 0.4 (range: 20–72 yrs); height 166 ± 0 cm; and weight 75.2 ± 0.6 kg) or ~1.5% of the total population >19 yrs underwent a range of health and fitness variables before intervention. Of the original sample, 310 were still training after 18 wks (42%) and a total of 151 subjects (age 43.6 ± 0.7 (range: 20–66 yrs); height 166 ± 1 cm; and weight 74.5 ± 1.1 kg) performed physical tests after intervention. The tests were performed during two prearranged test trials at each training venue before intervention and two test trials after intervention. Of the 310 participants active during the entire intervention period, 151 performed the tests both before and after intervention. The players not tested were not present during test trials before and/or after intervention. The recruitment was carried out through advertising via the web, social media, the media, and personal communication. The only exclusion criterion was a minimum age of 18 yrs and that the participants were mobile enough to take part in football play.

### 2.2. Experimental Design

In cooperation with the Faroese Football Association, all football clubs in the Faroe Islands were contacted and asked to organize football training for the local population. A total of 15 football clubs volunteered to take part in the study. A Football Fitness instructor was prepared for each club, and he/she organized the training during the intervention period, recording training attendance and injury frequency. During the 18 wks, 2–4 training sessions per week were organized, depending on the number of participants in the club. Training attendance was on average 1.6 ± 0.2 sessions (range: 0.6–2.9) per week, corresponding to 28.8 ± 1.0 training sessions in total during the 18 wks. The training was conducted as small-sided games (3v3 to 8v8), as previously described [25], and was preceded by a 10 min warm-up. Every session lasted for 1 h in total and heart rate was monitored during one random training session during the intervention period to describe the physical loading of the training. Prior to the training intervention and after 18 wks of training, the subjects were tested in a range of physical tests and had their blood pressure, resting heart rate, and body mass measured.

### 2.3. Experimental Procedures

Before and after the training intervention, the participants performed the Yo-Yo Intermittent Endurance test level 1 (Yo-Yo IE1) according to procedures described by Bangsbo and Mohr [26]. Heart rate was monitored during the test to determine maximal and submaximal heart rates (see, e.g., [27]) using Polar Team 1 heart rate monitors (Polar Electro, Kempele, Finland). The participants also completed the Arrowhead Agility Test (AAT) as described by Bangsbo and Mohr [26] where sprinting time was measured using photocell gates with a precision of 0.001 s placed 1 m above the ground (Newtest Powertimer System, Oulu, Finland). Finally, the Flamingo Balance Test (FBT) was conducted on left and right leg according to standard procedure [3]. All participants were familiarized with the testing procedures before the preintervention tests were completed, and all tests were performed by the researchers, who are highly experienced in all testing procedures used. Moreover, the participants were instructed to perform no physical training the day prior to the tests or on test days and to consume a similar diet the day prior to and on the testing day. Also, participants were asked to refrain from caffeine intake on the day of testing and from alcohol 24 h prior to testing. Both test rounds were conducted at the same time of the day (±1 h). After resting in a supine position for 20 min, blood pressure and resting heart rate were measured using automatic blood pressure monitors (HEM-709, OMRON, IL, USA). Five measurements were performed and the mean value was used as the test result. During one random training session, heart rate was recorded using Polar Team 1 heart rate monitors (Polar Electro, Kempele, Finland) in order to evaluate the intensity of the training.

### 2.4. Statistical Analyses

Data are presented as means ± SEM. Pre- and postintervention data were analyzed using one-way ANOVA. When a significant interaction was detected, data were subsequently analyzed using a Newman-Keuls* post hoc* test. The significance level was *P* < 0.05.

## 3. Results

### 3.1. Age and Gender Distribution

Of the 741 original participants, 310 were still taking part in the training after 18 wks, giving a success rate for the intervention of 42%. Of the original sample assessed before intervention (*n* = 502), 478 were women and 24 were men. The distribution in age groups 20–39, 40–59, and +60 yrs was 197, 290, and 12 participants, respectively, corresponding to 39.5, 58.1, and 2.4%. In age groups 20–39 and 40–59 yrs for women, 3.9 and 4.7% of all women in the Faroe Islands in these respective age groups took part in the intervention. The age distribution among participants completing the second test round (*n* = 151; Table 1) in age groups 20–39, 40–59, and +60 yrs was 50, 93, and 5 participants, corresponding to 34, 63, and 3%, respectively. The age distribution in the group of participants dropping out (Table 1) was 41, 57, and 2% for age groups 20–39, 40–59, and +60 yrs. The participants completing the intervention were 4.3% older (*P* < 0.05) than the dropout group (43.6 ± 0.7 versus 41.7 ± 0.4 yrs, resp. (Table 1)). This was the only recorded difference between the two groups (Table 1).

### 3.2. Cardiovascular Effects of the Intervention

**Figure 1:**
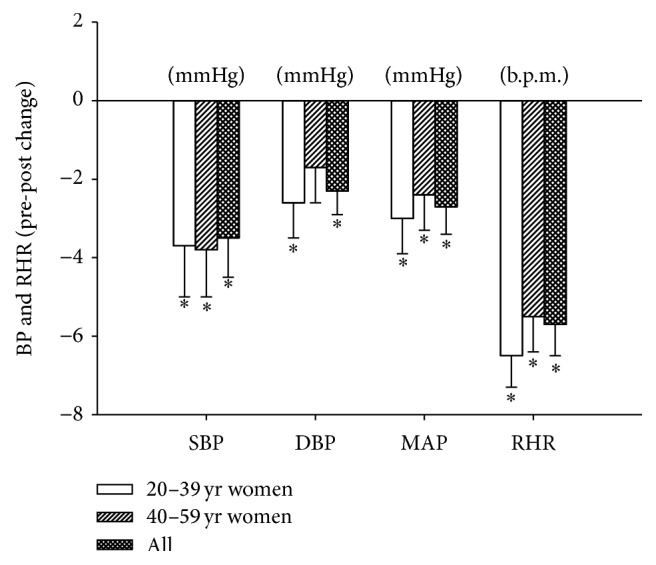
Changes scores in systolic blood pressure (SBP), diastolic blood pressure (DBP), and mean arterial pressure (MAP) as well as resting heart rate (RHR) after 18 wks of football training. Data are shown for all participants (*n* = 151) and women aged 20–39 (*n* = 51) and 40–59 (*n* = 88) yrs. Data are means ± SE. *∗* denotes a significant difference from preintervention. Significance level is *P* < 0.05.

**Figure 2:**
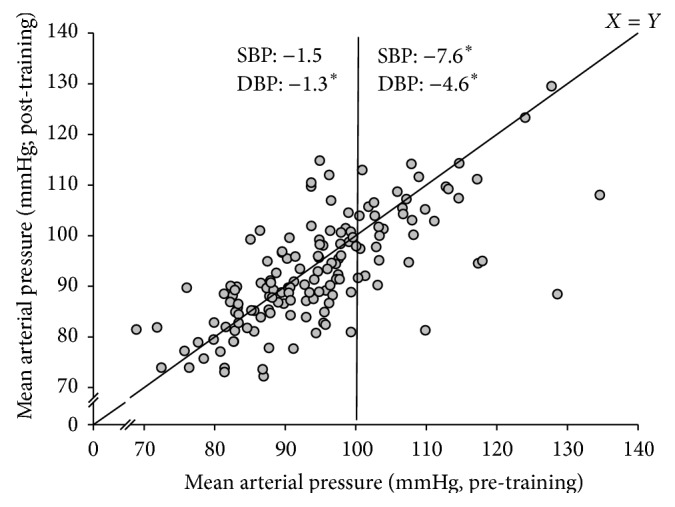
Individual mean arterial pressure (MAP; mmHg) before and after training (*n* = 151). Participants with pretraining MAP values >100 mmHg (*n* = 50) are separated by the vertical line from participants with MAP values <100 mmHg (*n* = 101). Pre- and post-values are separated by a line of identity. The change scores in SBP and DBP for the two groups are shown at the top of the figure. *∗* denotes a significant difference from preintervention. Significance level is *P* < 0.05.

SBP and DBP were 124 ± 1 and 81 ± 1 mmHg at baseline and decreased (*P* < 0.05) by −3.5 ± 1.0 and −2.3 ± 0.6 mmHg, respectively, during the 18 wk training period (Figure 1). For women in age groups 20–39 and 40–59 yrs, the reductions (*P* < 0.05) in MAP were −3.0 ± 0.9 and −2.4 ± 0.9 mmHg, respectively (Figure 1). In participants suffering from arterial hypertension (MAP > 100 mmHg; *n* = 50), there were marked reductions in MAP (−6 ± 1 mmHg; Figure 2) and the decrements in SBP, DBP, and MAP were greater (*P* < 0.05) than in the remaining normotensive participants (MAP < 100 mmHg; *n* = 101; Figure 2).

Resting heart rate was 77 ± 1 bpm at baseline but was lower (*P* < 0.05) by 8% (71 ± 1 bpm) after intervention (Figure 1). Women in age groups 20–39 and 40–59 yrs displayed a decrement (*P* < 0.05) in resting heart rate of 8.2 and 7.5%, respectively. Moreover, participants deemed to be hypertensive (MAP > 100 mmHg) had a decline (*P* < 0.05) in resting heart rate of 5 ± 1 bpm, which was not different from the decline (−6 ± 1 bpm; *P* < 0.05) in normotensive participants (MAP < 100 mmHg). Maximal heart rate determined at the end of the Yo-Yo IE1 test was 185 ± 1 bpm. Baseline heart rate after 1, 2, and 3 min of Yo-Yo IE1 was 157 ± 1, 167 ± 2, and 172 ± 2 bpm, corresponding to 84.9 ± 0.8, 90.3 ± 1.7, and 93.0 ± 1.8%  HR_max_, respectively. However, postintervention heart rate during the respective time-points was 151 ± 1, 165 ± 1, and 168 ± 2 bpm or 81.6 ± 1.6, 89.2 ± 1.7, and 90.8 ± 1.7%  HR_max_, which was lower (*P* < 0.05) at all time-points compared to preintervention.

### 3.3. Physical Capacity Effects

**Figure 3:**
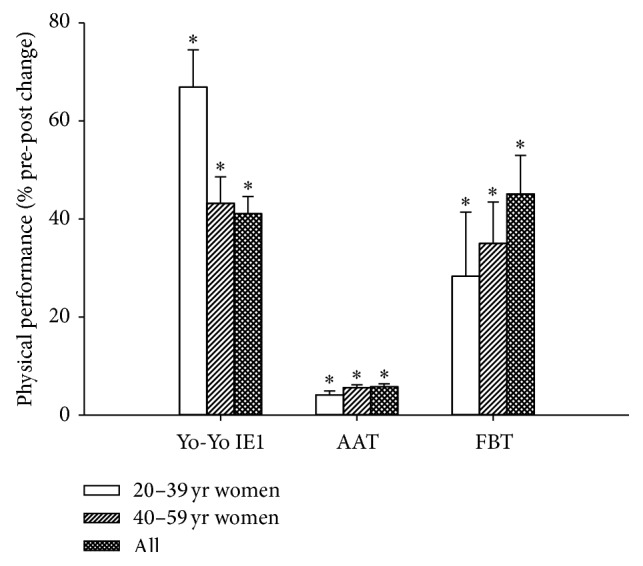
Change scores in Yo-Yo Intermittent Endurance level 1 test (Yo-Yo IE1), Arrowhead Agility Test (AAT), and Flamingo Balance Test (FBT) performance after 18 wks of football training. Data are shown for all participants (*n* = 151) and women aged 20–39 (*n* = 51) and 40–59 (*n* = 88) yrs. Data are means ± SE. *∗* denotes a significant difference from preintervention. Significance level is *P* < 0.05.

**Figure 4:**
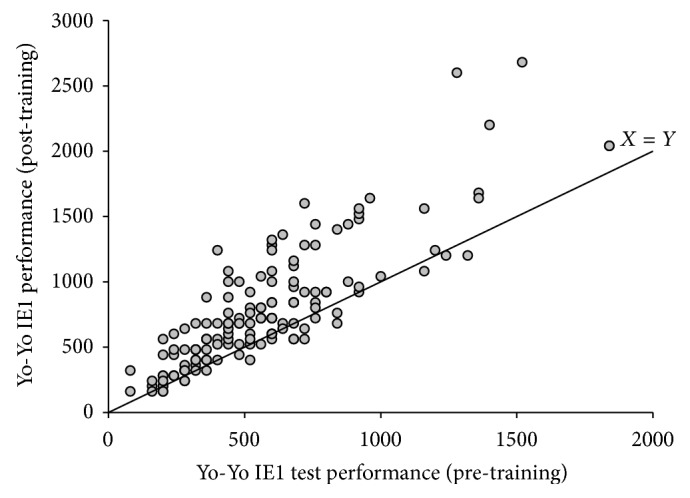
Individual Yo-Yo Intermittent Endurance test level 1 (Yo-Yo IE1) performance before and after training (*n* = 151) separated by a line of identity.

Yo-Yo IE1 performance was 540 ± 27 m before intervention and higher (*P* < 0.05) by 41% (752 ± 45 m) after intervention for the entire sample (Figure 3). The improvement in Yo-Yo IE1 performance was obtained irrespective of preintervention training status (Figure 4). AAT performance was 23.85 ± 0.30 s at baseline but was improved (*P* < 0.05) by ~6% (22.37 ± 0.21 s) (Figure 3). The number of falls during the Flamingo Balance Test on average for both legs was 4.1 ± 0.5 at baseline but decreased (*P* < 0.05) by 1.8 ± 0.3 falls after intervention (Figure 3).

### 3.4. Body Mass Effects

Body mass was 74.6 ± 1.1 kg at baseline but decreased (*P* < 0.05) by 0.8% to 74.0 ± 1.0 kg after intervention. The body mass reduction (*P* < 0.05) in women in age groups 20–39 and 40–59 yrs was 0.4 ± 0.2 and 0.7 ± 0.2 kg, respectively.

### 3.5. Physiological Loading during Training

Average and peak heart rates reached during training (*n* = 102) were 76.6 ± 0.7 (range: 67.6–92.4) and 96.8 ± 0.4 (85.7–100)%  HR_max_. This corresponded to 144 ± 2 and 183 ± 2 bpm in average and peak heart rate, respectively. Time spent in heart rate zones >80 and >90%  HR_max_ was 21.1 ± 1.1 (3.5–46.2) and 12.7 ± 1.3 (0.0–33.6) min, respectively, corresponding to 35.2 ± 2.1 and 21.2 ± 1.3% of the training time. There was no difference in cardiovascular loading during training in women aged 20–39 versus 40–59 yrs, nor in hypertensive versus normotensive participants (data not shown).

## 4. Discussion

For the first time, a large-scale and nationwide exercise intervention study involving more than 1.5% of the total population of a country and as high as 5% for middle-aged women has been completed. The major findings were as follows: (i) the recruitment was highly successful, especially among young and middle-aged women; (ii) attendance rate and exercise intensity were very high for recreational football training organized in local football clubs; (iii) adherence was moderate with 42% of the participants being active during the entire intervention period; and (iv) there were large fitness and health effects of this type of exercise intervention, which means that large-scale Football Fitness projects appear to be a valid tool for health promotion in inactive adult populations.

In small-scale societies, such as the Faroe Islands with only 50,000 inhabitants, it is feasible to initiate nationwide exercise intervention studies, such as the current intervention, due to relatively short geographical distances and communication infrastructure. Indeed, more than 2% of the adult population was recruited over a 2-month recruitment phase. Nearly all the participants were women, and of the 502 participants assessed before intervention, 95% were women. Thus, recreational football appears to be a popular leisure activity among women, which is supported by participation in the Football Fitness initiative organized in Danish football clubs, where 75% were women [24]. One reason for the appeal of Football Fitness for women may be that in Scandinavia a large proportion of men are already active in football clubs as well as self-organized indoor football. In relation to age groups, nearly 60% of the participants were middle-aged (40–59 yrs) women, while more than one-third were young women (20–39 yrs). This corresponds to 4 and 5% of all the women in the Faroe Islands in the respective age groups taking part in the nationwide Football Fitness intervention. It was observed that the adherence was moderate with 42% of the original sample still active after 18 wks. A figure that is almost similar to what has been observed in other health interventions such as “physical activity on prescription” where the adherence was 47% in a study with 366 untrained adults (66% women) that were enrolled in structured, supervised training combined with individual motivating conversations with a fitness instructor [28] is presented. With regard to the dropouts it should be noted that the only statistical difference between participants completing the intervention and the dropouts was that the dropouts were 4.3% younger. Future studies should aim at investigating the mechanisms determining adherence.

Of more than 300 participants still taking part in the intervention after 18 wks, 151 completed the fitness tests and other assessments before and after intervention. More than 90% of these were young and middle-aged women aged 20–66 yrs. It was demonstrated that systolic and diastolic blood pressure were lowered by 4 and 2 mmHg, respectively, for the entire sample after 18 wks of training, with no difference in change scores in mean arterial pressure between young and middle-aged women. Similar observations have been apparent in several randomized controlled trials of recreational football for women [10] and men [2, 25]. Moreover, for the 50 participants in the present study deemed to be hypertensive (MAP > 100 mmHg), MAP was lowered by as much as 6 mmHg, with greater effects than in the remaining normotensive participants (see Figure 2), which is also in accordance with previous football training studies involving hypertensive women [5] and men [4, 11]. A recent meta-analysis analyzing 123 studies involving more than 600,000 participants concluded that there were relative risk reductions for major cardiovascular diseases, such as coronary heart disease, stroke, and heart failure, proportional to the magnitude of blood pressure reduction achieved [29]. Moreover, the authors concluded that every 10 mmHg reduction in systolic blood pressure led to a 13% reduction in all-cause mortality. This was apparent with similar proportional risk reductions in trials with high and low mean baseline systolic blood pressure [29]. In the present study, despite the fact that the hypertensive group showed a larger effect than the normotensive group, the normotensive participants still had a significant drop of around 2 mmHg in mean arterial pressure after the training period. Thus, it is possible to organize a nationwide exercise intervention that lowers mean arterial pressure in both hypertensive and normotensive individuals. Since arterial hypertension is a major risk factor in relation to cardiovascular diseases [30] and is considered to be the most important risk factor for mortality and disability worldwide, causing nearly 10 million deaths per year [29, 31], the present intervention can be evaluated as a highly efficient health-promoting and rehabilitation initiative for the general population.

Resting heart rate has been shown to be an independent marker of cardiovascular risk in, for example, hypertensive individuals, and that increased RHR is linked to increased vascular oxidative stress, endothelial dysfunction, and accelerated atherosclerosis [32]. In the present study, resting heart rate was reduced by 8% (−6 bpm), which is in the same magnitude as shown in randomized controlled studies involving 3-4 months of football training in women [5] and men [2, 33]. The lowering of RHR was irrespective of age group or preintervention MAP. Moreover, after 1, 2, and 3 min of Yo-Yo IE1 test running, heart rate was markedly reduced. This indicates a lowered sympatic activity at rest and an elevated stroke volume during exercise, confirming previous football training studies [5, 10]. During training, average and peak heart rates were 77 and 97%  HR_max_, demonstrating a large cardiovascular loading, which is likely to have caused the markedly improved cardiovascular health profile of the participants. Indeed, time spent in heart rate zones >80 and >90%  HR_max_ was as high as 21 and 13 min per training session, respectively, which is similar to [1, 5] or slightly less than observed in conventional research studies with recreational football [25]. Despite a large range in cardiovascular loading among the participants, this was irrespective of age or preintervention cardiovascular health profile.

Body mass was reduced by 0.6 kg after the training intervention, which is less than the 1-2 kg reductions observed in other studies of similar duration [2, 5]. For example, Mohr et al. [5] studied middle-aged women, who had a body mass loss of 1.4 kg after 15 wks of training, compared to 0.7 kg in the present study for middle-aged women. One reason for this may be the lower training attendance in the present study, which was 1.6 sessions per week versus 3 sessions per week in the study by Mohr et al. [5]. In support of this notion is the fact that participants with high training attendance (>1.6 sessions per week; average 1.9 sessions per week) had a larger reduction in body mass (1.2 kg) compared to participants with low training attendance (<1.6 sessions per week; average 1.2 sessions per week), who reduced their body mass by 0.4 kg. Thus, high training attendance and corresponding higher total energy expenditure may induce the respective differences. However, the intervention was efficient enough to cause a significant weight loss, and the reduction in body fat is likely to be markedly higher, since studies of similar duration have shown a 1-2 kg gain in muscle mass based on whole-body DXA scans [2, 5, 34]. A limitation in the present study is the fact that diet was not controlled, which may have had an impact on the body mass alterations.

The Faroese Football Fitness intervention markedly increased the physical capacity of the participants. The endurance capacity was shown to be elevated 41% as evaluated with the Yo-Yo IE1 test. This is a change score of a magnitude in line with findings in comparable randomized controlled trials [4, 5, 34]. The improvement in Yo-Yo IE1 performance was obtained irrespective of preintervention training status. Thus, despite the fact that the participants had very different starting levels, the intervention induced an improvement in their endurance exercise capacity (see Figure 4). One reason for the increase is likely to be a higher aerobic capacity, indicated by the decrement in submaximal heart rates. Furthermore, several studies have demonstrated an elevated aerobic power as shown by an increased maximal oxygen uptake after 3-4 months of recreational football training [35]. A meta-analysis by Milanović et al. [16] of the effects of recreational football training revealed an average increase in maximal oxygen uptake of 3.51 mL/kg/min after short-term interventions comparable to the present study. Moreover, studies of muscular responses to football training in women have shown a marked increase in muscle oxidative capacity [36, 37], which may have contributed to the large increase in endurance exercise capacity in the present intervention.

For the first time, agility was tested before and after an intervention involving recreational football. An improvement of ~6% was observed in Arrowhead Agility Test performance, and this improvement in agility may be caused by an increased muscle mass, rate of force development, running speed, and neural drive, as also speculated in football studies with untrained individuals showing improvements in sprint performance [33] and jump performance [35]. In addition to improved agility, postural balance was markedly improved after the 18 wk intervention, and the magnitude of the change score confirms other comparable studies [38, 39]. The elevated postural balance combined with improved agility is highly relevant adaptations, since the majority of the participants were middle-aged women with a low physical capacity, who are a high risk group in relation to risk of bone fractures [40]. Since poor physical fitness is an independent cause of noncommunicable diseases [17], the intervention can be considered effective at inducing a great improvement in the general health profile of the participants. It is also noteworthy that the observed broad-spectrum fitness and health benefits occurred with an average training frequency of 1.6 per week and total training time of 96 min per week which is less than the recommendations from the World Health Organization (150 min/week) and from American College of Sport Medicine (3–5 times/week). However, several previous studies have shown marked positive effects of small-volume (2 × 15 min per week [14]) and medium-volume training (1.7–1.9 hours/week [4, 10]), indicating that less is needed when the training intensity is sufficiently high. Thus, it appears that several markers of health profile can be improved by low-volume exercise training, if high intensity exercise is included.

This is the first time that the effects of the Football Fitness concept have been evaluated on a nationwide scale in a real-life setting with the aim of promoting physical activity. Worldwide, 31% of all adults are inactive, with figures as high as 43% in some Western counties [41]. Inactivity rises with age and is higher in women than in men [41] and higher in high-income than in low-income countries [42]. Thus, exercise interventions targeting the adult population in the Western world, especially women, and an evaluation of the success rate in relation to recruitment, attendance, adherence, exercise intensity, and health effects are warranted.

In conclusion, the Football Fitness concept, when organized as a nationwide health-promoting intervention, is a relatively successful intervention that has a very high recruitment rate among women but also many dropouts. Moreover, the Football Fitness intervention was shown to be an effective health-promoting intervention with high exercise intensity, improvement in the cardiovascular health profile, and marked broad-spectrum elevations in the physical capacity of participants. We therefore recommend large-scale football training interventions as an alternative initiative for stimulating inactive individuals to take up an active lifestyle. Future studies should elucidate the social and motivations mechanisms governing the participation in health orientated exercise training intervention such as Football Fitness.

## Figures and Tables

**Table 1:** Characteristics of participants completing the training intervention (upper panel) and participants dropping out during the intervention period (lower panel).

Age (yrs)	Height (cm)	Weight (kg)	YYIE1 (m)	SBP (mmHg)	DBP (mmHg)
Participants completing the 18 wk intervention (*n* = 151)					
43.6 ± 0.7^*∗*^	166 ± 0	74.4 ± 1.0	586 ± 28	124 ± 1	81 ± 1
(20–66)	(152–185)	(45.1–109.5)	(80–1840)	(87–174)	(60–117)

Participants dropping out during the 18 wk intervention (*n* = 351)					
41.7 ± 0.4	166 ± 0	74.6 ± 0.8	544 ± 18	121 ± 1	80 ± 1
(20–67)	(146–192)	(46.1–174.6)	(80–2200)	(86–205)	(56–117)

*∗* denotes significant difference from participants dropping out. Significance level is *P* < 0.05.
